# A new therapy for drug-resistant malaria using Plasmodium synthetic lethality inference

**DOI:** 10.1186/1475-2875-11-S1-P61

**Published:** 2012-10-15

**Authors:** Eunseok Seo, Sang Joon Lee

**Affiliations:** 1 Center for Biofluid and Biomimic Research, Division of Integrative Biosciences and Biotechnology, Pohang University of Science and Technology (POSTECH), San 31, Hyoja Dong, Namgu, Pohang 790-784, Korea

## Background

If one gene of the *Plasmodium* (*P.*) synthetic lethal (SL) gene pair has antimalarial drug resistance (ADR), a drug targeting the other gene of the SL pair can be used as an effective antimalarial drug for treating drug-resistant strains of malaria diseases. The approach introduced in this study is based on bioinformatic database integration. It employs a promising concept of synthetic lethality and has practical applicability in identifying gene targets to discover potential future drugs.

## Materials and methods

Potential antimalarial drug target genes were identified by integrating whole biological information from many databases, including BioGRID [1], KEGG SSDD database [2], GeneDB [3], PlasmoDB [4], and Ensembl [5]. The representations of the resulting networks were constructed by using Cytoscape (version 2.8.1) [6]. The distribution networks on gene ontology annotation (GOA) terms were statistically analyzed using BINGO 2.44 Cytoscape plugging [7]. The fitness data on the yeast homologous genes of the finally selected *P.* genes were searched to find new drugs for clinical malaria treatment from Yeast Fitness DB [8].

## Results

A simple computational tool to analyze inferred SL genes of *P.* species (*P. falciparum*, *P. vivax and P. berghei*) was established to identify SL genes that are possible drug targets. Information on SL gene pairs with ADR genes and their first neighbors from the yeast SL gene was inferred to search for pertinent antimalarial drug targets. We suggested that specific antimalarial drug candidates can be inferred by searching drugs that cause a fitness defect in the yeast SL gene.

## Conclusions

We suggest a new concept of drug-resistant malaria therapy in this study. The alternative antimalarial drug therapy consists of data integration and inference through the homology analysis of yeast-human-*P.* Malaria parasites can be killed by mutating or blocking the SL partner of ADR genes. This concept is useful in selecting candidates as drug targets in antimalarial therapy. The methodology also provides not only drug target gene candidates for further experimental validation, but also information on new usage for already-described drugs. Drug candidates for targeting the suggested genes significantly benefit experimental validation in antimalarial drug discovery.
